# Galvanic Replacement of Electrochemically Restructured Copper Electrodes with Gold and Its Electrocatalytic Activity for Nitrate Ion Reduction

**DOI:** 10.3390/nano8100756

**Published:** 2018-09-25

**Authors:** Ali Balkis, Jessica Crawford, Anthony P. O’Mullane

**Affiliations:** 1 School of Science, RMIT University, Melbourne, VIC 3001, Australia; s3235954@student.rmit.edu.au; 2 School of Chemistry, Physics and Mechanical Engineering, Queensland University of Technology (QUT), Brisbane, QLD 4001, Australia; jessica.crawford@hdr.qut.edu.au

**Keywords:** galvanic replacement, electrocatalysis, nitrate reduction, nanostructures, active sites, hydrous oxides

## Abstract

The electrochemical formation of nanostructured materials is a cost effective route to creating substrates that can be employed in a variety of applications. In this work the surface of a copper electrode was electrochemically restructured in an alkaline solution containing ethanol as an additive to modify the surface morphology, and generate a Cu/Cu_2_O surface, which is known to be active for the electrocatalytic reduction of environmentally harmful nitrate ions. To increase the activity of the nanostructured surface it was decorated with gold prisms through a facile galvanic replacement approach to create an active Cu/Cu_2_O/Au layer. The surface was characterized by scanning electron microscopy, energy dispersive X-ray spectroscopy, X-ray photoelectron spectroscopy, as well as electrochemical techniques. It was found that the presence of recalcitrant oxides, and Au was beneficial for the increased activity compared to unmodified copper and undecorated restructured copper and was consistent with the incipient hydrous oxide adatom mediator model of electrocatalysis. This approach to generating nanostructured metal/metal oxide surfaces that can be galvanically replaced to create these types of composites may have other applications in the area of electrocatalysis.

## 1. Introduction

The electrochemical formation of nanostructured surfaces that are active electrocatalysts has received significant attention [1,2,3,4,5,6]. This is due to their wide-ranging applicability in diverse areas, such as energy conversion, energy storage, sensing, water treatment, additive manufacturing and electro-organic synthesis. There are significant advantages with such an approach whereby the size, shape and composition of the nanostructured surface can be controlled under ambient conditions using relatively straightforward instrumentation [1,6,7,8]. These factors can be determined through carefully choosing experimental parameters, such as applied potential, time, type of waveform, electrolyte composition and the substrate employed. One of the most common approaches to creating nanostructured surfaces is via the electrodeposition of materials from ionic salts on to a surface. An alternative method is to electrochemically pretreat or restructure a surface with the desired composition to increase surface area, active sites or induce some oxide formation.

This was highlighted very effectively for the case of Pt where square wave repetitive potential cycling in the presence of ascorbic acid was shown to generate highly active Pt that contained high-index facets on the surface [9]. It has also been shown that Pt nanoparticles change their size and shape in electrochemical environments, due to dissolution/deposition, oxide growth/reduction, and reconstruction [10]. This phenomenon has been taken advantage of for other metals, which have been restructured by applying oxidation/reduction protocols that initially form an oxide on the surface followed by reduction. The frequency and duration of the oxidation/reduction processes influences the morphology, number of active sites and hence electrocatalytic activity and has been demonstrated for Au and Cu surfaces [11,12,13]. Our recent work on the electrochemical restructuring of copper indicated that the presence of additives in the electrolyte can significantly influence the morphology of the surface and the extent of residual oxides, which impacted on electrocatalytic performance [13].

Copper has recently attracted interest for a variety of electrochemical applications other than the traditional smooth electroplating required for the semiconductor industry [14]. Copper can be activated electrochemically, via cathodic polarization in the hydrogen evolution region, to improve activity for hydrazine oxidation and nitrate reduction [15]. Nanostructured copper has also been used for the oxygen evolution reaction [16], electrochemical CO_2_ reduction [17,18], and the conversion of nitrate to ammonia [19]. In addition, the oxides of copper can also demonstrate good electrocatalytic activity, such as Cu_2_O for the oxygen reduction reaction [20], as well as a Cu/Cu_2_O/CuO electrode for the oxygen evolution reaction [21]. Therefore, creating nanostructured surfaces of copper with the presence of some oxides may offer an interesting route to active surfaces.

In electrocatalysis, bimetallic electrodes have been studied extensively, due to the synergy often seen when two metals are involved in a reaction [22,23,24,25,26,27,28]. Copper has also been alloyed with a variety of metals, such as Au, Ag, Pd and Pt to increase its (electro)-catalytic activity [29,30,31,32,33,34,35]. A relatively simple way to make bimetallic electrodes is via galvanic replacement [36,37,38,39,40] where for example copper acts as the sacrificial metal, which oxidizes, and transfers electrons to a metal salt in solution, which is subsequently reduced at the surface. This approach has been used to create many copper based bimetallic electrodes with interesting properties [41,42,43,44]. However, this work has also been extended to the galvanic replacement of metal oxide surfaces, such as Cu_2_O to create Cu_2_O/M (M = Pt, Pd, Au) materials that have potentially excellent applications [45,46,47,48,49,50].

In this work we investigate the electrochemical restructuring of a copper foil electrode in an alkaline electrolyte containing ethanol to generate a nanostructured surface containing residual surface oxides. This electrode was then galvanically replaced with Au to create a Cu/Cu_2_O/Au surface that was investigated for its electrocatalytic activity towards the reduction of nitrate ions. The latter was chosen due the environmental impact that nitrates have on lakes, rivers and streams, such as eutrophication and the potential for human health effects, such as liver damage [19,51]. The electrochemical removal of nitrate from water has been proposed as a viable means to alleviate this environmental concern [52], which has advantages over currently employed methods based on biological and physicochemical methods that are costly and more time consuming than an electrochemical method. Previous work has shown that copper is an effective sensing layer for the electrochemical detection of nitrate [53,54] and nitrite ions [53]. In addition, a recent example for nitrate and nitrite ion detection has also been reported using a CuNi alloy [55] indicating the benefits of a bimetallic electrode system for this particular analyte.

## 2. Materials and Methods

### 2.1. Chemicals

All chemicals were analytical grade reagents and used as received without further purification. Aqueous solutions of ethanol, HAuCl_4_, NaOH and KNO_3_ (Aldrich, Castle Hill, Australia) were made up with deionized water (resistivity 18.2 MΩ cm) purified by use of a Milli-Q reagent deionizer (Millipore, Australia). Cu foil with a thickness of 0.025 mm, and purity of 99.98% trace metals basis (Aldrich), was used as the substrate.

### 2.2. Sample Preparation

The restructured Cu electrode was fabricated using a cyclic voltammetry protocol (20 cycles recorded at 10 mV s^−1^ from −1.5 to 0.5 V versus Ag/AgCl) in a solution containing 0.1 M ethanol in 1M NaOH. The restructured surface was washed thoroughly with Milli-Q water and dried with nitrogen gas before the galvanic replacement reaction.

The gold decorated sample was achieved by immersing the restructured Cu foil into an aqueous solution of 5 mM HAuCl_4_ for 1–5 min at 22 ± 2 °C, after which it was thoroughly washed with Milli-Q water and blown dry with a stream of nitrogen.

### 2.3. Electrochemical Measurements

Cyclic voltammetry and differential pulse voltammetry experiments were conducted at 22 ± 2 °C using a CH Instruments (CHI 760C, Austin, TX, USA) potentiostat in an electrochemical cell (BAS, West Lafayette, IN, USA) that allowed reproducible positioning of the working, reference, and counter electrodes, and a nitrogen inlet tube. The copper electrodes were masked off using Kapton tape to give a defined area of 0.126 cm^2^ and were rinsed with Milli-Q water, acetone, and then in ethanol, and dried with nitrogen gas. The reference electrode was a Ag/AgCl (aqueous 3 M KCl) (BAS). For all of the electrochemical experiments, an inert graphite rod (with a diameter of 5 mm, Johnson Matthey Ultra ‘F’ purity grade) was used as the counter electrode to prevent any possible contamination from dissolution of the counter electrode [56]. Nitrate reduction experiments were performed using linear sweep voltammetry from −0.8 to 1.6 V at 100 mVs^−1^ in order to avoid further oxide formation before the experiment.

### 2.4. Surface Characterization

Scanning electron microscopy (SEM) measurements were performed on a FEI Nova scanning electron microscope equipped with an AMETEK energy dispersive X-ray (EDX) system (Nova 200, FEI, Hillsboro, OR, USA) operating at an accelerating voltage of 30 kV. X-ray photoelectron spectroscopy (XPS) analysis was performed using a Thermo K-Alpha instrument (Hillsboro, OR, USA) at pressures better than 10^−9^ Torr, with the data being referenced to the adventitious C 1s binding energy of 285 eV.

## 3. Results and Discussion

### 3.1. Preparation and Characterization of the Activated Electrode

**Figure 1:**
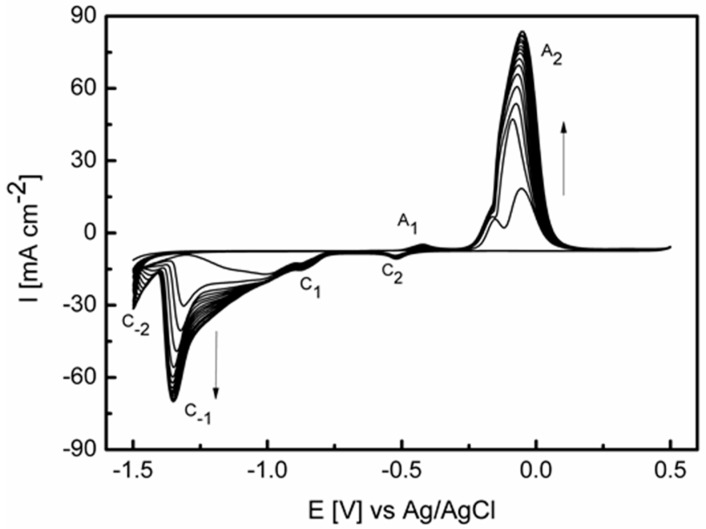
Repetitive cyclic voltammograms (20 cycles) recorded at a Cu foil electrode in 1 M NaOH containing 1 M ethanol at 10 mV s^−1^.

The electrochemical restructuring of a copper foil electrode involves oxidizing the surface followed by reduction. Illustrated in Figure 1 are 20 cyclic voltammograms that encompasses these processes when performed in 1 M ethanol and 1 M NaOH. The overall magnitude of the response increases with cycling (as indicated by the arrows showing the increasing current response), which indicates an increase in the surface area of the electrode. It should be noted that this increase is less than that observed when the Cu foil is restructured in 1 M NaOH only (Figure S1), as seen in our previous work [13], due to the presence of ethanol in the electrolyte and is discussed below. On the anodic sweep peak A_1_ is due to the Cu/Cu_2_O transition followed by process A_2_, which is split into two components namely oxidation of unoxidized Cu^0^ and Cu_2_O to CuO and Cu(OH)_2_. Upon cycling the two processes merge into a dominant peak with a slight shoulder and can be related to a decrease in the amount of Cu^0^ at the surface of the electrode. On the cathodic sweep process C_2_ is the reverse of process A_2_. However the magnitude of the response is significantly lower, as the reduction of CuO or Cu(OH)_2_ results in the formation of a passive film of Cu_2_O, which is significantly more difficult to reduce. This transiently formed Cu_2_O is then reduced in a broad response indicated by C_1_. This behavior is typical of the electrochemistry of copper electrodes in alkaline electrolyte [15,57,58,59]. Upon cycling there is the emergence of new processes C_−1_ and C_−2_, which are attributed to recalcitrant hydrous oxide species. Cathodic peaks at these potentials have been achieved previously for thermally treated copper electrodes or those polarized in the hydrogen evolution region for prolonged periods [57,60]. It is interesting that the presence of ethanol in the electrolyte results in an oxidation/reduction process that initiates the formation of these hydrous oxides on the surface of the copper, which are reduced over different potential ranges compared to Cu foil restructured in 1 M NaOH only (Figure S1). This indicates that ethanol is perturbing the oxide growth/reduction process as seen previously when phenylacetic acid and benzyl alcohol were used as additives [13]. Recent work has shown that the presence of ethanol in the electrolyte during Co(OH)_2_ electrodeposition significantly increases the supercapacitor performance of the material compared to when it was deposited from a water only electrolyte [61]. The electrodeposition of Ni-Co hydroxides from ethanol-water solutions also has been demonstrated to generate a material with enhanced properties for the oxygen evolution reaction [62]. Hydroxyl ion-assisted alcohol reduction of copper complexes to metallic copper has also been reported [63], which is similar to the electrolyte conditions employed here and indicates the role that ethanol can play in the growth of metal nanoparticles, as well as metal oxide/hydroxide materials.

**Figure 2:**
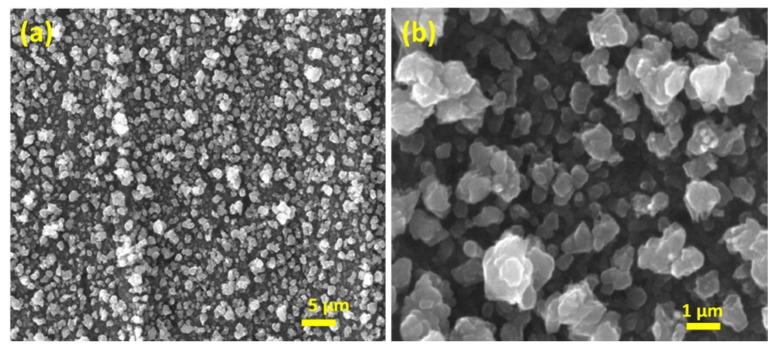
Scanning electron microscopy (SEM) images at (**a**) low, and (**b**) higher magnification, for a Cu foil electrode restructured in 1 M NaOH containing 1 M ethanol at 10 mV s^−1^ for 20 cycles over the potential range of −1.5 to 0.5 V.

The surface of the electrode was then investigated by SEM, and is shown in Figure 2. After the electrochemical treatment, the surface of the foil is significantly rougher than the pristine sample (Figure S2). Upon closer inspection the surface consists of clusters of micrometer sized particles where each cluster consists of agglomerated smaller particles (Figure 2b). The open and quite porous structure is also consistent with the increased magnitude of the current upon repetitive cycling (Figure 1) whereby access to the analyte is maintained during film growth and its reduction. From the low magnification image the restructuring process is quite homogeneous across the entire surface of the electrode (Figure 2a).

**Figure 3:**
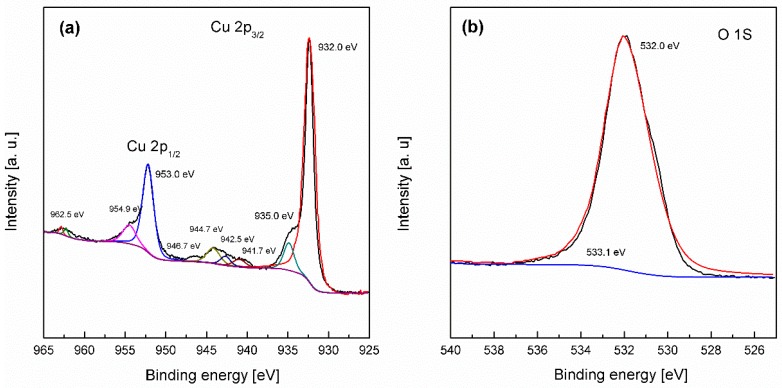
X-ray photoelectron spectroscopy (XPS) spectra for (**a**) Cu 2p_3/2_ and (**b**) O 1s for a Cu foil electrode restructured in 1 M NaOH containing 1 M ethanol at 10 mV s^−1^ for 20 cycles over the potential range of −1.5 to 0.50 V.

To ascertain the chemical composition of the surface and whether residual oxides are present, X-ray photoelectron spectroscopy (XPS) was undertaken (Figure 3). The Cu 2p spectrum shows a Cu 2p_3/2_ peak at 932.0, which is consistent with the presence of Cu or Cu_2_O and a peak at 934.9 eV, as well as a series of shakeup satellite peaks from 941 to 944.7 eV, which is consistent with the presence of Cu^2+^, such as Cu(OH)_2_ and CuO on the surface of the electrode [64]. The broad O 1s spectrum centered at 532.0 eV is consistent with Cu(OH)_2_ formation with some evidence of adsorbed H_2_O by the minor feature at 533.1 eV [64]. This data is consistent with the cyclic voltammetry data (Figure 1), which showed the presence of C_−1_ and C_−2_ processes, which in turn were attributed to the reduction of oxides on the surface. The composition of oxides on the surface of copper after electrochemical activation is complex and is also likely to consist of hydrous oxides and not simply the ones assigned here. It also suggests that electrochemical reduction does not entirely remove these oxides from the surface of the copper during repetitive potential cycling, which is also observed by eye by the extensive discoloration of the copper surface after cycling.

As mentioned in the introduction the galvanic replacement of Cu, as well as Cu_2_O is readily achieved and was investigated by immersing the restructured sample in a solution of 5 mM HAuCl_4_. Cu can be galvanically replaced with Au via the following:

3Cu(s) + 2AuCl_4_^−^_(aq)_ → 2Au_(s)_ + 3Cu^2+^_(aq)_ + 8Cl^−^_(aq)_ (1)

whereas Cu_2_O is replaced by gold via [46]:

2AuCl_4_^−^_(aq)_ + 6H^+^_(aq)_ + 3Cu_2_O_(s)_ = 6Cu^2 +^_(aq)_ + 8Cl^−^_(aq)_ + 2Au_(s)_ + 3H_2_O_(l)_ (2)

**Figure 4:**
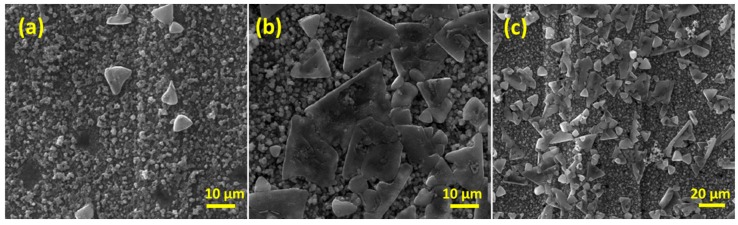
SEM images for a restructured Cu foil (as in Figure 2) galvanically replaced with gold via immersion in an aqueous solution of HAuCl_4_ for (**a**) 1 min, (**b**) 3 min and (**c**) 5 min.

For the latter it indicates that three protons are required for every AuCl_4_^−^ ion to achieve the stoichiometric ratio of Au(III)Cl_4_^−^:H^+^. Therefore, to promote Au formation on Cu_2_O, the solution should be acidic to force reaction 2 to the right. This is ensured when 5 mM of HAuCl_4_ is used for the galvanic replacement process [48]. Figure 4 shows SEM images of the restructured copper electrode after galvanic replacement with gold using 5 mM HAuCl_4_ for 1, 3 and 5 min. After 1 min of reaction there is evidence of large prism shaped deposits on the surface of the electrode. EDX analysis of the sample confirmed the presence of Au (Figure S3). Upon increasing the reaction time to 3 min the sizes of the prisms increase, as well as the coverage on the surface. This trend continues after 5 min of galvanic replacement, which results in a surface decorated extensively with gold prisms of various sizes. The formation of prism like deposits may be due to the liberation of halide ions, namely chloride ions, into solution during the galvanic replacement process, as denoted in Equations (1) and (2). Previous work has demonstrated that chloride ions liberated from AuCl_4_^−^ play a major role in promoting the growth of (111) oriented triangular gold nanoparticles through traditional chemical synthesis routes [65]. It should also be considered that at the interface where the concentration of chloride ions will be high, that [CuCl_2_]^−^ species may also form [66], which ensures the solubility of the liberated copper ions and may influence the growth process.

**Figure 5:**
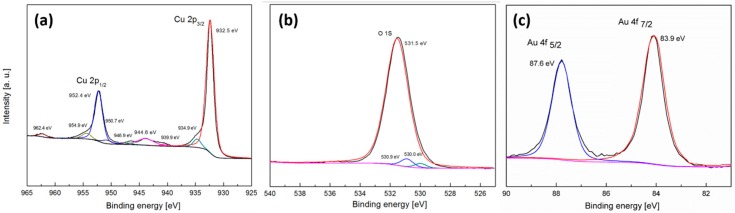
XPS spectra for (**a**) Cu 2p_3/2_, (**b**) O 1s and (**c**) Au 4f for a restructured Cu foil electrode galvanically replaced with gold via immersion in HAuCl_4_ for 3 min (i.e., for the sample shown in Figure 4b).

The formation of gold was further confirmed by XPS analysis where the Au 4f spectrum shows peaks at binding energies of 83.9 for Au 4f_7/2_ and 87.6 eV for Au 4f_5/2_, which is consistent with metallic gold [67]. The Cu 2p and O 1s spectra remain relatively unchanged compared to the restructured electrode (Figure 3) and indicates that any Cu^2+^ species that may have been formed during galvanic replacement are expelled into solution consistent with the formation of [CuCl_2_]^−^ species and not on the surface, which would have been accompanied by an increase in intensity of the peak at 934.3 eV in the Cu 2p spectrum. It should be noted that there is a minor component at 530 eV in the O 1s spectrum (Figure 5b), which indicates the presence of some CuO on the surface, however it is negligible in comparison to the other copper species.

### 3.2. Nitritate Ion Electrocatalysis

**Figure 6:**
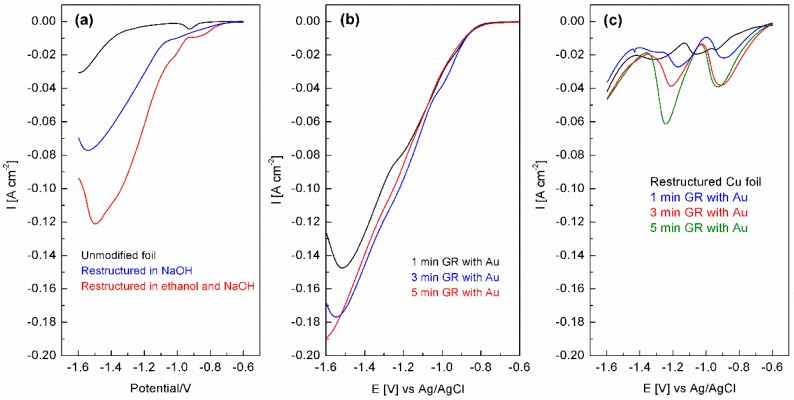
Linear sweep voltammograms recorded at 100 mV s^−1^ in 1 M NaOH in the presence of 0.1 M KNO_3_ showing (**a**) the effect of electrochemical restructuring, (**b**) the effect of galvanic replacement of restructured Cu with gold and (**c**) the electrochemical behavior of these electrodes in the absence of KNO_3_.

The electrocatalytic activity for the restructured Cu foil and the galvanically replaced surface with gold was then investigated for nitrate ion reduction in 1 M NaOH. Initially the unmodified Cu foil, the Cu foil restructured in 1 M NaOH only and the Cu foil restructured with 1 M ethanol present in the electrolyte were compared. From Figure 6a it can be observed that the Cu foil electrode that had been subjected to repetitive potential cycling in the presence of ethanol showed significantly enhanced performance for nitrate ion reduction as evidenced by the less negative onset potential and increased current density over the entire potential range. The slight cathodic peak seen at ca. −0.90 V in all cases is due to the reduction of residual oxides on the surface. The increased activity could just be related to the increased surface area of the film compared to the Cu foil and therefore it should be noted that the data was normalized to the area of each electrode, which was calculated as follows. The charge passed for process A_2_ on the 1st cycle of a cyclic voltammogram for a bare Cu foil electrode in 1 M NaOH was equated to the masked off geometric area of the electrode (0.126 cm^2^). Therefore, for each modified electrode the charge passed for process A_2_ on the 20th cycle was used to estimate the area of the restructured electrode. Comparing the 1st cycle in Figure S1 with the 20th cycle in Figure 1 shows that there is not a significant change in the charge associated with process A_2_, in fact there is a slight decrease to give an area of 0.122 cm^2^ for the restructured electrode and indicates that the surface area is not particularly changed when additives, such as ethanol are added to the electrolyte bur rather its presence changes the nature of residual oxides on the surface. Nevertheless, the data presented here is still an indication of the specific activity of the electrodes used in this study.

Once the oxides are removed at ca. −0.90 V there is an increase in current corresponding to the reduction of nitrate to nitrite ions [64]. This is followed by a significantly larger increase in current at ca. −1.1 V, which is due to the reduction of nitrite ions formed in the first reduction process. When the surface is decorated with gold it has a major impact on the electrocatalytic performance of the electrode. For this work, the restructured electrode obtained with the addition of ethanol to the electrolyte was used for the galvanic replacement process and electrocatalytic activity for nitrate reduction. The presence of gold results in a further shift in the onset potential to less negative values, which is also accompanied by an increase in current density for the entire potential range (Figure 6b). The amount of gold plays a role in the activity whereby the activity increases from 1 min of galvanic replacement to 3 min, but 5 min of reaction time does not result in any improvement. The gold coverage estimated from EDX analysis for the 3 min of galvanic replacement gave a value of 9 wt %. This synergistic effect between Cu_2_O and Au, as well as the bimetallic Cu/Au system has been reported previously for heterogeneous catalysis reactions [33,46,47,68,69], as well as electrocatalytic reactions [33,70,71,72]. To ensure that the electrocatalytic response was not due to the reduction of residual oxides on the surface the same linear sweep voltammetry experiments were performed in the absence of nitrate ions (Figure 6c). Here the magnitude of the responses is significantly lower than that seen during catalysis. However, the reduction of oxides will contribute to the overall current seen in Figure 6b, up to ca. −1.2 V. From the data in Figure 6c there are clearly two main reduction processes at −0.9 and −1.2 V. The onset potential for the reduction of the oxides coincides with the increases in catalytic current, seen in Figure 6b, for nitrate to nitrite reduction and then the further reduction of nitrite ions. This type of behavior is consistent with the Incipient Hydrous Oxide Adatom Mediator (IHOAM) model of electrocatalysis proposed by Burke [73,74]. Reduction of recalcitrant oxides on metal surfaces expose active metallic sites, in this case copper sites (Cu*), which are responsible for the reduction of nitrate ions. Once the nitrate ions receive an electron from Cu* it is oxidized into a hydrous oxide, however, due to the potential of the electrode, it is quickly reduced back to Cu* thereby enabling the electrocatalytic reduction of nitrate to continue. This in essence, sets up a surface confined redox mediator couple (Cu*/Cu hydrous oxide), which mediates the electrocatalytic reaction. From the data in Figure 6c there are two distinct Cu*/Cu hydrous oxide couples at −0.9 and −1.2 V, which are even more pronounced in the presence of gold and may be the origin of the increased electrocatalytic performance of the gold decorated Cu/Cu_2_O surface compared to the restructured Cu/Cu_2_O electrode. This data is akin to an electrochemically activated copper electrode via cathodic polarization in the hydrogen evolution region previously reported by Burke and Bond who reported the presence of active site responses at −0.95 and −1.10 V vs. Ag/AgCl in 1 M NaOH using large amplitude Fourier transform ac voltammetry, which mediated the reduction of nitrate ions and the oxidation of hydrazine [15]. It should be noted that the composition of these hydrous oxides is complex and has been postulated to consist of a variety of species, such as Cu_2_O, Cu(OH)_2_, Cu(OH)·H_2_O and CuO(OH^−^)_0.5_ [59]. Therefore, the reduction of these species will lead to a variety of Cu* active sites that would consist of adatoms or clusters of adatoms. In addition, during the electrocatalytic reaction these Cu* sites will be created in the presence of the Au prisms that decorate the surface. This will access the synergistic effect between Cu and Au thereby also providing a means to increase activity. Control experiments performed using Cu foil that was galvanically replaced with gold under the same conditions did not yield any increase in nitrate ion reduction current density (Figure S4). The amount of gold deposited was 14 wt %, which is greater than that for the restructured surface indicating that the presence of Cu_2_O on the surface of the foil is less reactive towards the galvanic replacement process. The presence of Au prisms however was consistent (Figure S5) indicating that the growth mechanism is the same at both surfaces. These observations indicate that the presence of a Cu/Cu_2_O/Au interface is responsible for the increased electrocatalytic activity and that the reduction of oxides at the surface that expose Cu* sites plays a significant role.

**Figure 7:**
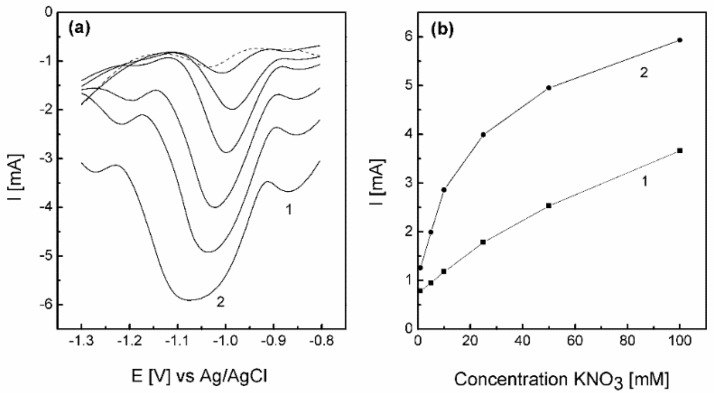
(**a**) Differential pulse voltammetry recorded for restructured Cu galvanically replaced by Au for 5 min in 1 M NaOH containing 0 (dashed line), 1, 5, 10, 25, 50 and 100 mM KNO_3_, and (**b**) a plot of peak height versus concentration for peak 1 and peak 2 identified in (**a**).

To further clarify that the response recorded is due to nitrate reduction and not just residual oxides a concentration study was undertaken over the range of 1 to 100 mM NO_3_^−^. Differential pulse voltammetry was utilized (Figure 7a) to give a better analytical signal. Interestingly, the nitrate to nitrite response, which is evident as a shoulder at ca. −0.95 V in Figure 6b when linear sweep voltammetry was used, is enhanced to give a well-defined peak (labelled peak 1). This is followed by further reduction of nitrite at ca. −1.05 V, which shifts negatively as the concentration is increased, due to uncompensated resistance effects. For process 1 and 2 the peak heights were plotted as a function of concentration, and are shown in Figure 7b. In both cases the response is linear up to 10 mM, but then gradually deviates from linearity, which is not unsurprising at these high concentrations. This indicates that in principle this surface could be employed as a sensing layer for nitrate detection, as well as a possible electrocatalyst for the remediation of nitrate from waste water streams.

## 4. Conclusions

The electrochemical restructuring of Cu foil is readily achieved using a simple potential cycling protocol that involves the oxidation of the surface followed by reduction. The presence of ethanol was found to perturb this process and modify the surface to generate a porous morphology in contrast to the relatively smooth pristine Cu foil. The restructured electrode contained residual oxides, which were unable to be reduced during the fabrication process, and were found to contain Cu_2_O species. This Cu/Cu_2_O electrode was then galvanically replaced with gold to create gold prisms on the surface. The electrocatalytic activity of the electrodes was investigated for the reduction of nitrate ions where the following trend was found; Cu foil < restructured Cu foil in NaOH < Cu foil restructured with ethanol additive < Au decorated restructured foil using the ethanol additive. The presence of residual oxides and gold was found to be a key aspect for the increased activity. The reduction of the residual oxides during the electrocatalytic reaction to expose Cu* sites in the presence of metallic gold resulted in improved performance for nitrate ion reduction, which is consistent with the IHOAM model of electrocatalysis. This combination of restructuring metal electrodes followed by galvanic replacement may open up other combinations of metal/metal oxides that have electrocatalytic activity.

## Supplementary Materials

The following are available online at http://www.mdpi.com/2079-4991/8/10/756/s1, Figure S1: SEM image of Cu foil, Figure S2: cyclic voltammograms showing the restructuring of Cu foil in 1 M NaOH, Figure S3: EDX analysis of restructured foil decorated with Au, Figure S4: cyclic voltammograms shoing the reduction of nitrate ions at a Cu foil decorated with gold and Figure S5: SEM image of Cu foil decorated with gold.
