# Supplementary material for: Galvanic Replacement of Electrochemically Restructured Copper Electrodes with Gold and Its Electrocatalytic Activity for Nitrate Ion Reduction
Source: Nanomaterials (Basel). 2018 Sep 25;8(10):756. doi: 10.3390/nano8100756 (PMC6215138; doi:10.3390/nano8100756)
Supplement: Supplementary file 1 [file nanomaterials-08-00756-s001.pdf]

nanomaterials

Galvanic Replacement of Electrochemically Restructured Copper Electrodes with Gold and its Electrocatalytic Activity for Nitrate Ion Reduction

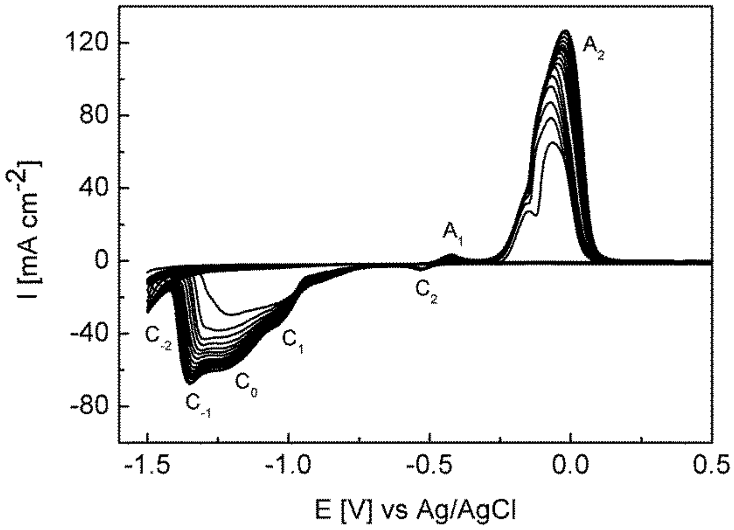

Figure S1. Repetitive cyclic voltammograms (20 cycles), recorded at a Cu foil electrode in 1 M NaOH at 10 mV s^{-1}.

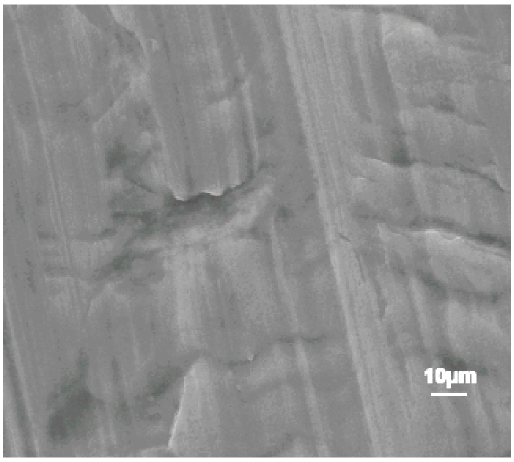

Figure S2. Scanning electron microscopy (SEM) image of an unmodified Cu electrode.

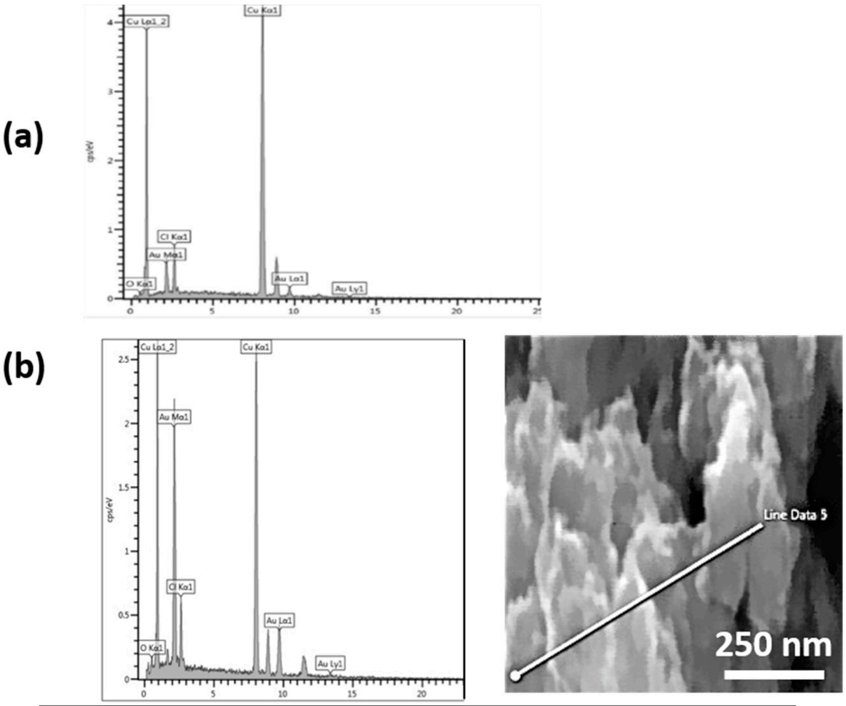

Spectrum label	Line sum spectrum
O	0.81
Cl	3.75
Cu	58.41
Au	37.03
Total	100.00

Figure S3. (**a**) EDX spectrum for a restructured Cu foil galvanically replaced with gold via immersion in an aqueous solution of $HAuCl_4$ for 3 min, and (**b**) a line spectrum taken through a deposit on the surface which indicates a high concentration of gold.

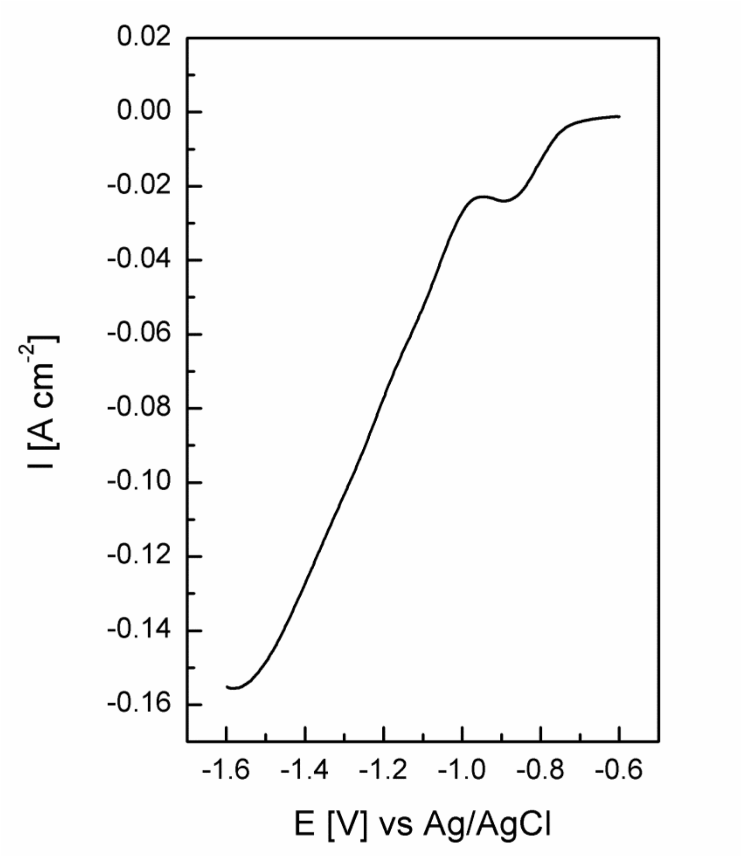

Figure S4. Linear sweep voltammogram recorded at 100 mV s^{-1} in 1 M NaOH in the presence of 0.1 M KNO_3 at a Cu foil electrode galvanically replaced with gold via immersion in an aqueous solution of $HAuCl_4$ for 3 min.

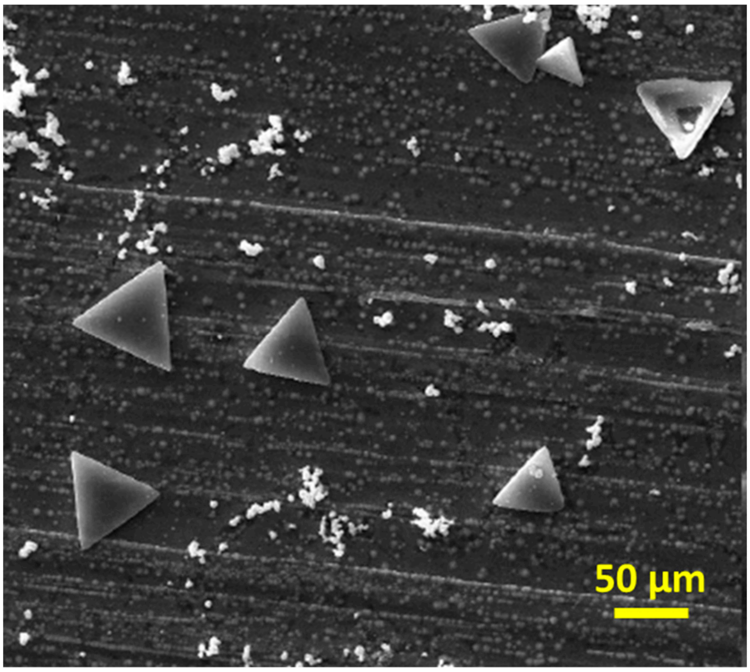

Figure S5. SEM image of a Cu foil electrode galvanically replaced with gold via immersion in an aqueous solution of $HAuCl_4$ for 3 min.
